# Factors Affecting Anti-Glycan IgG and IgM Repertoires in Human Serum

**DOI:** 10.1038/srep19509

**Published:** 2016-01-19

**Authors:** Saddam M. Muthana, Jeffrey C. Gildersleeve

**Affiliations:** 1 Chemical Biology Laboratory, National Cancer Institute, NIH, 376 Boyles St., Frederick, MD 21702, USA; 2 Chemistry Department, College of Science & General Studies, Alfaisal University, Riyadh, KSA

## Abstract

Serum anti-glycan antibodies play important roles in many immune processes and are of particular interest as biomarkers for many diseases. Changes in anti-glycan antibodies can occur with the onset of disease or in response to stimuli such as pathogens and vaccination. Understanding relationships between anti-glycan antibody repertoires and genetic and environment factors is critical for basic research and clinical applications, but little information is available. In this study we evaluated the effects of age, race, gender, and blood type on anti-glycan antibody profiles in the serum of 135 healthy subjects. As expected, IgG and IgM antibody signals to blood group antigens correlated strongly with blood type. Interestingly, antibodies to other non-ABH glycans, such as the alpha-Gal antigen, also correlated with blood type. A statistically significant decline in IgM signals with age was observed for many antibody subpopulations, but not for IgG. Moreover, statistically significant correlations between race and IgG levels to certain LacNAc-containing glycans were observed. The results have important implications for designing studies and interpreting results in the area of biomarker discovery and for the development of vaccines. The study also highlights the importance of collecting and reporting patient information that could affect serum anti-glycan antibody levels.

Human serum contains a diverse assortment of anti-glycan antibodies that play critical roles in immunology and provide a rich reservoir of potential biomarkers for many biomedical applications and diseases. The most well-known examples of anti-glycan antibodies are those that bind ABH blood group antigens and ones that bind xenoantigens such as the alpha-Gal antigen. Detection of anti-glycan antibodies against blood group A and B antigens provides a simple and reliable strategy to predict which individuals are suitable matches for transfusions and transplants^1^,^2^,^3^. Human serum contains numerous other anti-glycan antibody populations that are crucial in other areas of immunology, such as tumor surveillance, autoimmunity, defense against pathogens, and response to vaccines. As a result, there has been significant interest into exploring the potential use of circulating anti-glycan antibodies as biomarkers for wide variety of diseases including cancer^4^,^5^,^6^,^7^,^8^, Crohn’s disease (CD)^9^, multiple sclerosis (MS)^10^, type 1 diabetes mellitus^11^, neuropathy^12^, and peptic ulcers^13^.

Information about the factors that influence antibody repertoires is critical for both basic research and biomarker studies. Humans show large diversity in their repertoires of serum anti-glycan antibodies, but relatively little is known about the factors that generate and regulate this diversity. Antigen exposure accounts only partially for variations among individuals in their repertoire of serum anti-glycan antibodies. Anti-glycan antibodies can be produced to self or altered self-antigens and to antigens without known exposure. Therefore, many anti-glycan antibodies do not adhere to the paradigm of an adaptive immune response and are often referred to as “natural antibodies”^14^,^15^. Information about the factors that affect anti-glycan antibody diversity are essential for designing appropriate studies, analyzing results, and distinguishing disease-specific changes from normal variation between individuals. For example, biomarker discovery and validation is often carried out with the use of case-control studies, in which the anti-glycan antibody profiles of a group of patients are compared to control subjects. Knowing which traits account for variation in anti-glycan antibodies among individuals will help to identify differences between cases and controls that are disease specific. For example, antibody levels to blood group antigens can vary among healthy individuals with different blood types^16^. Imbalances in blood type distributions between cases and controls could bias certain antibody measurements. Age has also been reported to affect anti-glycan antibody profiles, but different studies have found inconsistent effects, including decreases with age^17^, increases^18^, and no correlation^19^. Therefore, more information on factors that contribute to variations of anti-glycan antibody levels are needed to properly design experiments and interpret results.

Glycan array technology provides a powerful high-throughput tool for studying the interactions between carbohydrates and macromolecules^20^,^21^,^22^,^23^,^24^,^25^,^26^. Glycan arrays allow one to profile serum antibody levels for hundreds of glycans in a single experiment using minimal amounts of precious clinical samples and expensive or scarce carbohydrates. Recently, glycan arrays have been used to identify serum anti-glycan antibody subpopulations with utility as biomarkers for variety of diseases^6^,^7^,^8^,^9^,^10^,^18^,^27^,^28^,^29^,^30^,^31^,^32^,^33^,^34^,^35^,^36^,^37^,^38^,^39^,^40^,^41^ and vaccines^42^,^43^. In this study, we used glycan array technology to evaluate changes in free serum anti-glycan and anti-glycopeptide IgG and IgM of 135 healthy subjects of varied age, race, gender, and blood type. We found that blood type, age, and race can have a significant effect on anti-glycan antibody populations. Our results help to resolve previous conflicting reports on the effects of age and provide important information for designing and interpreting studies in the area of biomarker research and vaccine developments.

## Results

### Experimental design

We profiled anti-glycan IgG and IgM antibody repertoires in a set of 135 serum samples from seemingly healthy individuals with variations of age, race, gender, and blood type (see Supplementary Information, Table SI) on a glycan microarray. The microarray was composed of neoglycoproteins, natural glycoproteins, and controls. The neoglycoproteins were produced by covalently attaching multiple copies of a glycan or glycopeptide onto a carrier protein, such as bovine serum albumin. While the linkage between the glycan/glycopeptide and protein is non-natural, neoglycoproteins can display glycans/glycopeptides at similar densities as one would find on natural glycoproteins. In addition, one can vary density by modulating the average number of glycans/glycopeptides per molecule of carrier protein. Neoglycoproteins have been used for many years in the field of glycobiology as multivalent probes/inhibitors in a variety of assays^44^. The array used in this study contained 330 different array components, including a diverse assortment of *O*-linked and *N*-linked glycans, glycans from glycolipids, non-human glycans, glycopeptides, and natural glycoproteins. These array components were selected to capture a wide spectrum of anti-glycan antibodies. A full list of array components and sequence information can be found in the Supporting Information.

A multiplexed array-based assay developed by our group was used to measure IgG and IgM simultaneously in a single experiment^45^. Sera were diluted 1:50 to ensure that the majority of antibodies are detected, while only a few components are at the spot saturation levels. Assays were carried out at 37 °C in order to evaluate antibody binding properties at physiological temperature. Low affinity, cold reactive anti-glycan antibodies may not be detected under these conditions. In addition, the assay is designed to measure free antibody; antibody-antigen complexes may not be detected. To minimize false-positives due to multiple comparisons and confirm findings, sera were divided into two sets (training set and validation set) and were analyzed independently. The samples in each set were selected to include a balanced representation for each category, rather than being proportional to the distribution in the general population. The training set (n = 45) is composed of 25 females, 20 males, 15 African Americans, 16 Caucasians, 14 Hispanics, 13 A-type, 12 AB-type, 5 B-type, 16 O-type, and median age = 35 years. It was first analyzed to assess variations in anti-glycan antibody levels in healthy individuals and to identify correlations with respect to age, race, gender, and blood type. All 45 samples in the training set were from Valley Biomedical Products and Services (Winchester, VA). Next, we analyzed the validation set (n = 90) to confirm results and validate the correlations found in the training set. The validation Set (n = 90) is composed of 45 females, 45 males, 34 African Americans, 30 Caucasians, 26 Hispanics, 29 A-type, 16 AB-type, 13 B-type, 32 O-type, and median age = 37 years.

To examine the effect of variations in sample collections on anti-glycan antibody profiles, the validation set included samples from two different sources (60 samples from Valley Biomedical Products and Services (Winchester, VA), and 30 samples from Bioreclamation LLC (Westbury, NY). The overall antibody profiles for the two sources were similar indicating no systematic differences due to sample collection or handling by the two providers. Correlations identified in the training set but not confirmed in the validation set were attributed to chance and were classified as false positives.

### Differences in anti-glycan antibody repertoires

**Figure 1:**
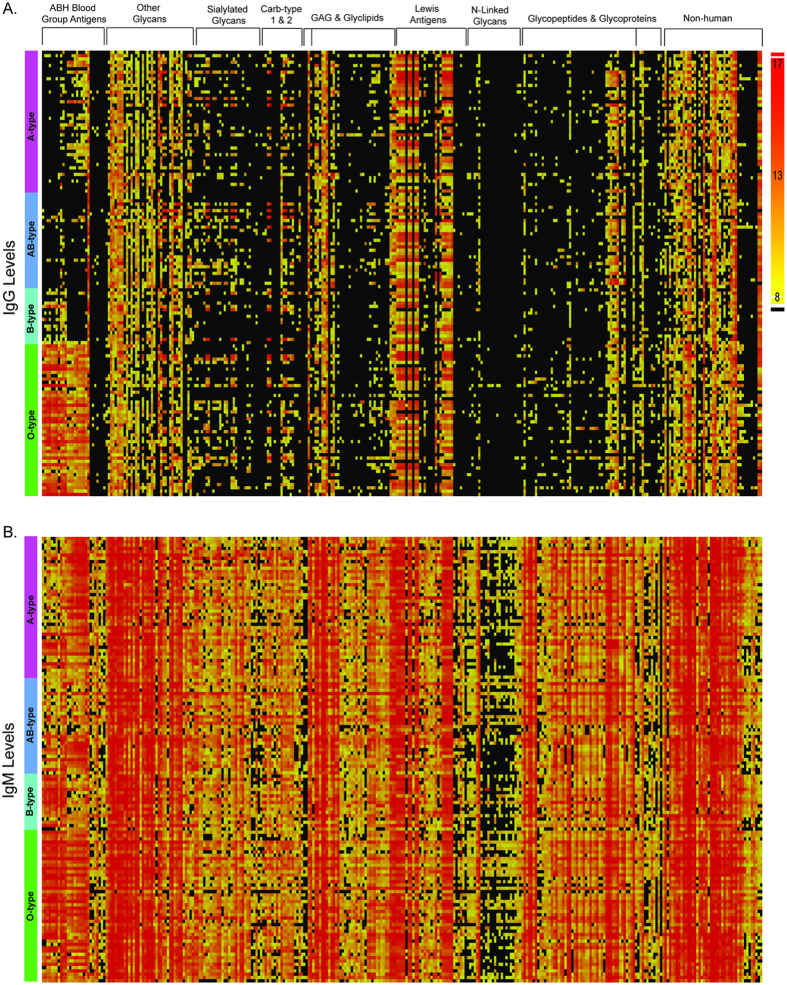
Heat maps of IgG (**A**) and IgM (**B**) signals from 135 healthy donors. Subjects are clustered by blood type in the rows. Glycans are organized into families in the columns. Black boxes indicate no measurable signal in our assay.

To provide an overall view of the anti-glycan antibody repertoires, heat maps of IgG and IgM anti-glycan antibody signals of seemingly healthy adults were constructed (Fig. 1). Samples were grouped by subjects’ blood types, and the glycans were clustered into families. In general, individuals had high IgG and IgM antibody signals to various glycans including many non-human glycans, Lewis C antigens, and some glycolipids and glycopeptides. Signals of IgG and IgM against blood group H antigens and *N*-linked glycans were consistently low.

**Figure 2:**
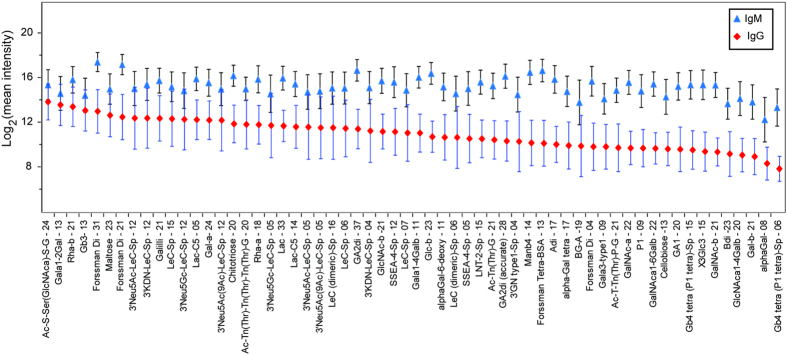
The distribution (log-transformed base 2) of IgG and IgM antibody signals for 60 array components with relatively high IgG and IgM signals.

Next, we compared the distribution of IgG and IgM antibody signals in both training and validation sets and found them to be very consistent. Figure 2 shows the distribution of IgG and IgM antibodies for selected array components that were consistently high in training and validation sets. Consistent with previous studies^17^,^29^,^46^,^47^, among the highest antibody signals observed were for alpha and beta rhamnose and the Forssman antigens (di- and tetrasaccharides). However, the highest and most consistent signals of IgG antibodies across all subjects in training and validation sets were to the glycopeptide Ac-Ser-Ser(GlcNAcα)-Ser-Gly, a previously unreported subpopulation of anti-glycopeptide antibodies. GlcNAcα linked to serine have been found in prokaryotes but not in human^48^. The IgG and IgM signals correlated with each other, but variability in IgG and IgM antibody signals existed for certain glycans. For example, IgM antibody signals for certain glycopeptides were relatively high, but the IgG signals for the same glycans were low.

### Blood type

The ABH blood group antigens are key glycans that define a person’s blood type. Individuals normally have serum antibodies that recognize blood group antigens that are not expressed on their own cells. Thus, blood type A and O individuals typically have antibodies to the blood group B antigen, blood type B and O individuals generally have antibodies to the blood group A antigen, and blood type AB individuals do not have antibodies to either blood group A or B antigens. While correlations between blood type and antibody levels to blood group antigens were expected^16^,^17^,^46^, several matters have not been addressed. First, it is unclear whether IgG or IgM antibodies would correlate better with blood type. Second, the terminal trisaccharides for blood group A [GalNAcα1-3(Fucα1-2)Galβ] and B [Galα1-3(Fucα1-2)Galβ] can be attached to various carrier glycan chains, and it is unclear whether variations to the carrier chains affect correlations with blood type. Third, little is known whether correlations between blood type and serum antibodies extend beyond the standard blood group antigens.

As anticipated, free IgG and IgM antibody signals to blood group A and B antigens showed statistically significant correlations with blood type (Table 1). IgG antibody signals to all the blood group A and B determinants correlated more strongly with blood type than IgM signals. For example, IgG signals to Globo A displayed a much stronger correlation with blood type (training set, median log_2_ signal = 7.20 for A and AB individuals vs. 13.00 for B and O individuals, *p* = 4.15 × 10^−15^) than IgM signals (training set, median log_2_ signal = 14.34 for A and AB individuals vs. 14.63 for B and O individuals, *p* = 7.59 × 10^−3^). Similar trends were observed with other blood group variants in the training and validation set. It is important to note that serum antibodies to blood group A and B did not correlate perfectly with blood type. For example, some AB individuals had high IgM signals to A-antigens, B-antigens, or both. Antibodies to self-antigens in healthy individuals are common, especially for natural antibodies (i.e. antibodies derived from B1 cells or their equivalent, often of the IgM isotype)^49^. These antibodies are thought to play a role in clearing cell debris/material from dead tissue/cells, although the exact roles are still being elucidated. Antibodies to self ABH blood group antigens have been detected previously in human serum^50^. Presentation of the blood group A and B determinants on a carrier chain substantially improved correlations with blood type. Blood group antigens displayed on type 1, 2, and 4 chains displayed much higher correlations with blood type than the terminal trisaccharides. For example, IgG signals to A tetrasaccharide type 2 (A tetra type 2-Sp-17, median log_2_ signal = 7.20 for A and AB individuals vs. 12.60 for B and O individuals, *p* = 2.54 × 10^−17^) correlated much stronger with blood type than blood group A trisaccharide (BG-A_tri_-19, median log_2_ signal = 7.50 for A and AB individuals vs. 13.05 for B and O individuals, *p* = 2.84 × 10^−7^). Similar trends were observed for blood group B tetrasaccharide versus the trisaccharide [for B tetra type 2-Sp-20, median log_2_ signal = 7.20 for B and AB individuals vs 11.00 for A and O individuals (*p* = 2 × 10^−11^); BG-B_tri_-13, median log_2_ signal = 7.20 for B and AB individuals vs 10.65 for A and O individuals (*p* = 2 × 10^−5^)]. Better correlations for tetrasaccharides than trisaccharides were also observed for IgM antibodies. As for the specific type of carrier chain, the best correlations were observed when the blood group determinants were displayed on type 2 chains for both IgG and IgM. This observation may be due to the fact that type 2 chains are the major carriers of ABH-determinants in erythrocytes. It should be noted, however, that the array used in this study did not contain type 3, 5 and 6 chains. Therefore, additional studies will be necessary to evaluate these variants.

Beyond the blood group antigens, we observed statistically significant correlations between blood type and anti-glycan antibody signals for certain non-ABH epitopes (Table 2). For example, IgG antibodies to the alpha-Gal tetrasaccharide (Galα1-3Galβ1-4GlcNAcβ1-3Galβ) and Bdi (Galα1-3Galβ) xenoantigens had a significant correlation with blood type in the training and validation sets (*p* < 0.02). Other significant associations with blood type were noticed for IgG signals to a variety of alpha-Gal-like determinants, the Forssman tetrasaccharide (GalNAcα1-3GalNAcβ1-3Galα1-4Galβ), and to the glycoprotein bovine submaxillary mucin (BSM). BSM has the blood group A antigen on it. The other glycans are distinct antigens but share the same terminal di- or trisaccharide backbone as that of blood group A or B antigens (See Supplementary Information, Figure S1). For example, the terminal trisaccharide (Galα1-3Galβ1-4GlcNAcβ1) of alpha-Gal tetrasaccharide is the same trisaccharide backbone as blood group B antigen type II. For IgM antibodies, correlations with a tripeptide containing the Tn antigen (Ac-S-Tn(Ser)-S-G, *P* < 0.01) and Adi (p < 0.02) were statistically significant in both training and validation sets. Additionally, associations with blood type were noticed for IgM signals to asialo ovine submaxillary mucin (OSM; >90% of the glycans are the Tn antigen) and GalNAcα1-6Galβ.

### Age

Aging is a complex process that is associated with a number of alterations in immune function (immunosenescence)^51^, including changes in immunoglobulin levels. Several studies reported age-associated variations in total antibody levels, although each Ig isotype changed differently^52^,^53^,^54^. The relatively few studies on the subpopulation of serum antibodies that bind carbohydrate antigens have yielded conflicting results^17^,^18^,^31^, possibly due to technical differences in the glycans and antibody isotypes studied. To shed additional light on these discrepancies, we sought to evaluate the effects of age on each isotype (IgG and IgM) separately.

**Figure 3:**
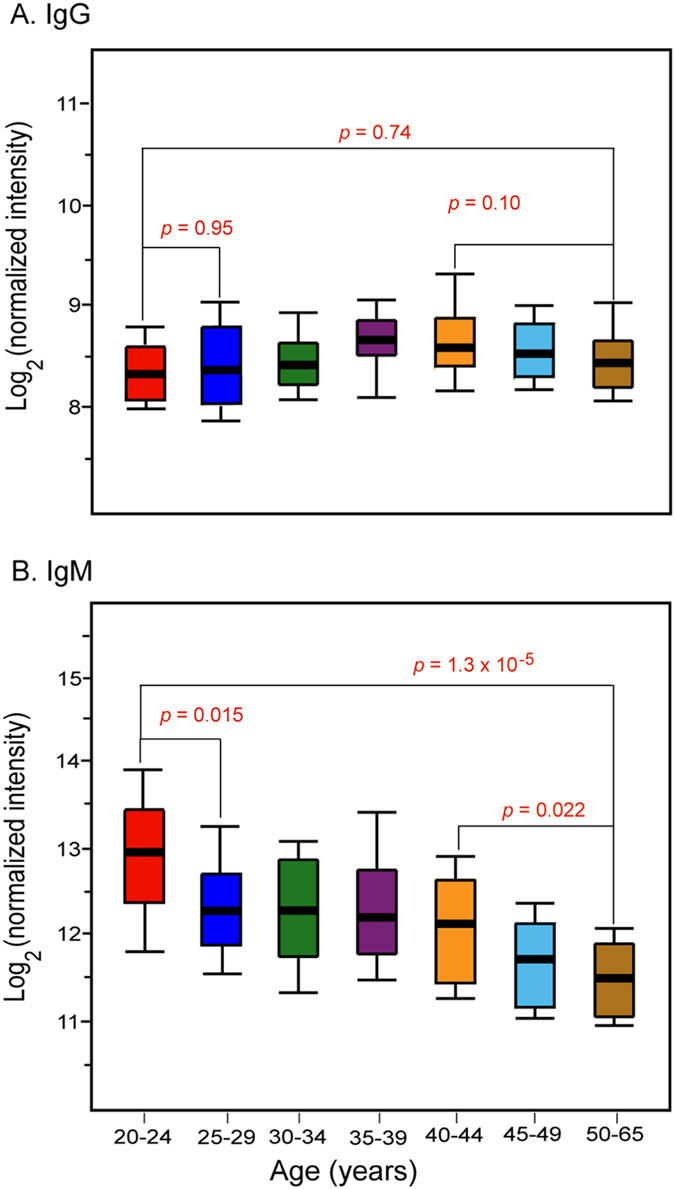
Variations in anti-glycan IgG and IgM antibody signals with age. (**A**) Box plots show minimum variation in anti-glycan IgG antibody signals. (**B**) Box plots of the decrease in IgM signals.

**Figure 4:**
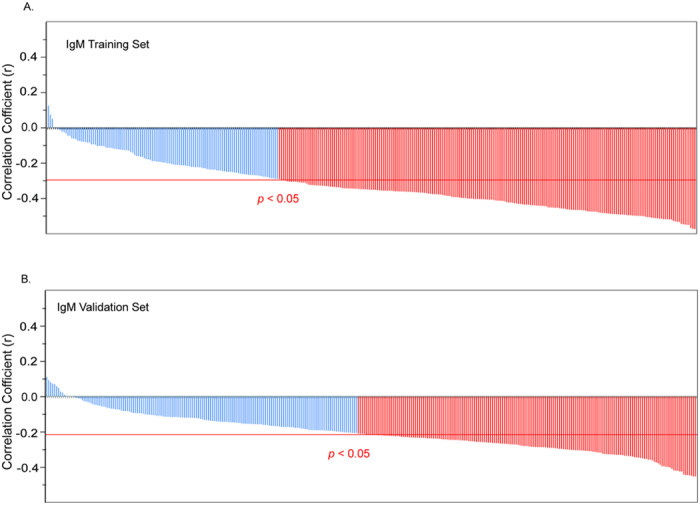
Age-related variations in anti-glycan antibodies in apparently healthy individuals. Bar graphs displaying correlation coefficients for all array components indicate a significant age-related decrease in IgM anti-glycan antibody signals for majority of the carbohydrates on the array in the training set (**A**) and validation set (**B**).

The effects of age on anti-glycan antibody profiles were quite different for IgG and IgM antibodies. The average IgG signals on the array for individuals remained relatively unchanged with age (See Supplementary Information, Figure S2A and Figure S3). From the different correlation tests performed, only the Spearman test showed a near significant aged-related increase (Pearson’s r = 0.16, *p* = 0.07; Spearman’s r = 0.17, *p* = 0.05; Kendall’s r = 0.12, *p* = 0.2). However, no individual glycan had a statistically significant correlation with age in both the training and validation sets for IgG. In contrast, average IgM signals showed a statistically significant age-related decrease in all the tests (Pearson’s r = −0.40, *p* = 1 × 10^−6^; Spearman’s r = −0.41, *p* = 8 × 10^−7^; Kendall’s r = −0.29, *p* = 7 × 10^−4^) (See Supplementary Information, Figure S2B and Figure S4). To illustrate this relationship, subjects were clustered into 7 groups based on age (5 year ranges for subjects aged 20–49 years plus a ≥50 year old group). For each group, box plots were generated based on the mean intensity over the entire array (Fig. 3). While no significant changes in IgG signals with age were observed (Fig. 3A), a statistically significant decrease in IgM signals with age was detected (Fig. 3B). Interestingly, anti-glycan IgM signals did not decline uniformly with age. The box plots show a statistically significant decrease in IgM signals (*p* < 0.015) from the first age group (20–24 yrs) to the second group (25–29 yrs), but signals remained relatively stable until the early 40s. Then, IgM signals started to decrease significantly in subjects aged 45 years and older. Similarly, a statistically significant decline in IgM signals was observed when subjects were grouped by decade of life. In both the training and validation sets, nearly all individual IgM signals decreased with increasing age, and more than half of these were statistically significant (*p* < 0.05) (Fig. 4). The declines in IgM signals were much greater than the expected 20% general reduction in in IgM levels with increasing age^52^,^53^,^54^,^55^. The mean IgM signal measured with our array decreased by 62% (*p* = 1.3 × 10^−5^) for the same age range (20–60 yrs).

### Gender

In addition to age, gender is an important factor that could affect anti-glycan antibody levels. In general, the average anti-glycan IgG signals measured on our array for all ages were statistically similar for both females and males (*p* = 0.97), but the average anti-glycan IgM signals were slightly higher in females (*p* = 0.13). Although not statistically significant, the trend observed for anti-glycan IgM is consistent with previous reports showing higher total IgM levels in females^53^,^54^,^55^,^56^. Moreover, we found that gender did not correlate consistently with measured IgG or IgM antibody signals to any specific glycan in training and validation sets. Therefore, our data suggest that gender does not seem to significantly affect the anti-glycan IgG or IgM repertoires.

*Race*. With respect to race, no statistically significant variations were observed in the average anti-glycan IgG (*p* = 0.15) or IgM (*p* = 0.14) signals measured on the array among all three races included in this study. However, we found that IgG signals for certain LacNAc-containing glycans correlated with race in both training and validation set (Table 3). In general, the highest IgG signals were observed among African-Americans and lowest among Caucasians.

## Discussion

Anti-glycan serum antibodies are a key element of the immune system and are a rich but underexplored source of potential biomarkers. Information about factors that influence serum levels of anti-glycan antibodies is crucial for understanding the roles of these antibodies and for exploiting them for clinical applications. In this exploratory study, we evaluated the effects of age, race, gender, and blood type on variability of free IgG and IgM anti-glycan antibody levels in seemingly healthy adults. A number of statistically significant correlations were uncovered, and the results could have broad implications in designing studies and interpreting results in different areas including biomarker research and the development of vaccines and immunotherapeutics. It is important to note that the vast majorities of glycans showed no correlation with race, gender, and blood type, and the average signals for individuals over the entire array were very similar. These results indicate that the observed correlations are not due to differences in total antibody levels.

One of the most interesting and important implications of this study is that blood type influences serum antibody subpopulations beyond the expected blood group antigens. It is well known that individuals possess antibodies to ABH blood group antigens that are not present within their body. In this study we found that serum antibodies to other, non-ABH antigens also correlate with blood type. For example, measured antibody signals to various alpha-Gal terminal antigens and to glycopeptides containing the Tn antigen showed statistically significant correlations with blood type. These relationships presumably arise as a result of structural similarity between the Tn antigen and BG-A and between the alpha-Gal antigen and BG-B. While structurally related to ABH antigens, these glycans are biochemically distinct. The Tn antigen is a tumor-associated carbohydrate antigen, and the alpha-Gal antigen is a xenogenic glycan expressed on a variety of non-human sources^57^,^58^,^59^. The correlations with blood type for these antibody subpopulations are relevant in several areas. First, serum antibodies to these glycans have been reported to have altered levels in cancer patients relative to healthy individuals, and they have been proposed as potential cancer biomarkers^5^,^60^. Since blood type influences antibody levels to these glycans, ABO blood type must be considered and controlled for when designing additional case-control studies to evaluate the potential of these antibody subpopulations as biomarkers.

Second, the correlations of Tn and alpha-Gal antibodies with ABO blood type have important implications for the development of vaccines and immunotherapeutics. The presence of pre-existing antibodies that bind to vaccine components can influence the ensuing immune response in a variety of ways. In certain cases, pre-existing antibodies can improve responses by enhancing antigen uptake and processing^61^,^62^. In other cases, pre-existing antibodies can capture and neutralize vaccine components rendering them ineffective^63^,^64^. There are a number of vaccines and immunotherapies in pre-clinical and clinical development that contain either the Tn antigen or the alpha-Gal antigen. For example, incorporation of the highly immunogenic alpha-Gal antigen into whole human cancer cell vaccines has been used as a strategy to break tolerance and enhance the immune response^65^,^66^. The whole cell vaccine algenpantucel-L, which is currently in Phase III clinical trials for the treatment of pancreas cancer, is a prime example of this approach^67^. The enhanced immunogenicity of these modified cells is due to recognition of the alpha Gal antigen by pre-existing serum antibodies. The alpha-Gal modification approach has been integrated into a variety of other immunotherapeutics as well^68^,^69^. The prevailing view is that IgG antibodies to alpha-Gal are consistently high in all humans^70^,^71^, suggesting that the alpha-Gal modification strategy should be applicable to most patients. Our results show a greater than 32 fold difference in anti-alpha-Gal IgG signals between the subjects in the 5^th^ percentile and 95^th^ percentile. Since there is considerable variability and anti-alpha-Gal IgG signals correlated with blood type, an unexpected implication of our results is that efficacy of alpha-Gal modified therapeutics may also be associated with blood type. Blood type may also influence efficacy of Tn-based cancer vaccines.

In addition to blood type, we also observed strong correlations between anti-glycan antibodies and age. In particular, IgM antibodies to many glycans decreased with increasing age, while IgG antibodies showed little or no alteration with age. The large decline in IgM signals for most glycans on the array helps explain why we previously observed age-related decreases in signals when measuring overall immunoglobulin (IgG, IgA, and IgM), whereas others did not see an age related decline for IgG signals. IgM antibodies to glycans generally are much more abundant than glycan-binding IgG and IgA. Therefore, the combined signal was dominated by IgM antibodies. In addition, our results indicate that age is a critical factor when designing case-control studies to identify serum anti-glycan antibodies as potential biomarkers, as one could observe differences in anti-glycan IgM levels between groups that are unrelated to disease/condition (false positives) if the groups are not properly matched for age (or the data are not normalized for differences in age). Finally, the relationship between IgM anti-glycan antibodies and age is also important for vaccines and other immunotherapies. For example, we have previously found that pre-treatment IgM levels to the blood group A antigen correlate positively with overall survival for prostate cancer patients treated with PROSTVAC-VF^43^. Since these antibody levels are anticipated to decrease in older patients, age will be an important factor to consider in the development of pre-treatment IgM as a biomarker for selecting patients likely to benefit from PROSTVAC-VF therapy.

Some differences in antibody signals were also observed based on race. In particular, differences in IgG antibody signals to certain LacNAc-containing glycans were statistically significant based on race. Interestingly, serum antibodies to some of these glycans have been reported to discriminate patients with malignant ovarian tumors from healthy controls^31^. Therefore, strategies to account for variations based on race will be important for future validation studies on these potential biomarkers. Although the race categories used here are broad and further studies are needed, the information obtained from this study is a positive first step toward understanding the impact of race on antibody repertoires.

In conclusion we demonstrated that age, race, and blood type can have a significant effect on anti-glycan antibody populations. Although, this study focused on a limited number of potential covariates, it highlights the importance of collecting and reporting patient information when developing biomarkers or immunotherapeutics that may be affected by serum anti-glycan antibodies. Oftentimes, key demographic information is not included in studies on anti-glycan antibodies. Without appropriate matching, inadvertent imbalances in these factors could increase the likelihood of false-positives or cause normal variation among individuals to mask disease-associated differences. Therefore, matching cases and controls is crucial when profiling anti-glycan antibody levels for biomarker discovery. Finally, there are other factors that could potentially influence anti-glycan antibody repertoires, such as smoking, season of blood draw, diet, and medications. Thus, additional studies are needed to more fully define the factors and traits that affect anti-glycan antibody populations.

## Materials and Methods

### Serum samples

Sera were purchased from Valley Biomedical Products and Services (Winchester, VA) or Bioreclamation LLC (Westbury, NY). Samples were tested in accordance with FDA regulations and found to be negative for HIV ½ AB, HCV AB, and non-reactive for HBSAG, HIV-1 RNA, HCV RNA, and STS. Sample processing and storage by both providers was carried out under standardize procedures. The procedures for the two companies were similar except for how whole blood was clotted and centrifuged to obtain serum (room temperature for Valley Biomedical Products and Services versus 4 °C for Bioreclamation LLC). The overall antibody profiles were similar for the two companies, indicating no systematic differences (see Supplementary Information, Figure S5). The 135 samples were obtained from 70 females and 65 males between the ages of 20 and 65 years. Donors were classified as African-American, Caucasian, and Hispanic and having blood types A, B, AB, and O (see subjects’ characteristics in Supplementary Information, Table SI). The majority of samples (105 out of 135) were collected from two geographical locations (Florida and Tennessee). All samples were stored at −80 °C until used.

### Array fabrication and binding assay

Serum samples were profiled on a glycan array containing 330 components including self-antigens, tumor-associated carbohydrate antigens, glycopeptides, glycoproteins, *N*-linked glycans, glycan portions of glycolipids, non-human glycans, and some controls (see Supporting Excel File). The carbohydrates were conjugated to BSA or HSA to produce neoglycoproteins (carbohydrates covalently coupled to proteins via a non-naturally occurring linkage) prior to printing. In addition to variations in structure, some glycans were printed at different densities by varying the average number of glycan molecules per molecule of protein carrier. The number following the name abbreviation refers to the average glycan density (number of glycans/protein carrier). Glycan density can have an enormous impact on the avidity and binding selectivity of antibodies, due to the ability of antibodies to form multivalent interactions^72^,^73^,^74^.

Glycan arrays were fabricated as previously reported^75^ except for the addition of a washable fluorescent dye to the print buffer as an indicator of successful liquid deposition and spot morphology (see Supplementary Information). The array format and assay have been described previously^76^, along with analysis of reproducibility^17^ and validation with numerous antibodies and lectins^77^,^78^,^79^,^80^. Prior to each experiment, slides were pre-scanned and then a 16-well module (Grace Bio-Lab) was affixed to the slide in order to create 16 independent array wells. Pre-scanned images were analyzed for technical faults and saved as a permanent record. The slides were blocked overnight at 4 °C with 3% BSA (w/v) in PBS and washed 6 times with PBST (PBS with 0.05% (v/v) Tween 20). All samples including reference sample (pooled serum from healthy donors) were diluted 1:50 in 3% BSA and 1% HSA in PBST, and then 100 μL of each sample was added into two different wells on different slides and allowed to incubate at 37 °C with gentle agitating (100 RPM) for 4 h. After washing 3 times with PBST, bound antibodies were detected by incubating with DyLight 549 goat anti-human IgG (Jackson ImmunoResearch 109-505-008) and DyLight 649 goat anti-human IgM (Jackson ImmunoResearch 109-495-043) in 3% HSA and 1% BSA in PBS for 2 h at 37 °C with gentle agitating. The anti-IgG reagent detects all IgG subclasses, and signals for IgM and IgG are roughly comparable on micrograms/mL level^45^. After washing 7 times with PBST, slides were removed from modules, immersed in wash buffer for 5 min, and centrifuged at 1000 rpm for 5 min. All clinical/demographic information was blinded during the profiling of serum samples.

### Image analysis and data processing

Slides were scanned at 10 μm resolution with a Genepix 4000A microarray scanner (Molecular Devices Corporation) and analyzed with Genepix Pro 6.0 software as previously reported^76^. The spots were defined as circular features with a diameter of 90 μm. The background-corrected median was used for data analysis, and technical faults (e.g., missing spots) were flagged and excluded from further analysis. To minimize the impact of noise on our comparisons, spots with intensity lower than 150 (1/2 the typical background signal when analyzing IgM at 1:50) were considered too low to be measured accurately and were set to 150. The average of duplicate spots was calculated and normalized to the reference samples. A log-transformed (base 2) was applied for each slide, and the final data value was obtained from the normalized average of data from both slides. Statistical analysis and correlations were obtained using Partek Genomics Suite software. Analyses of variance (ANOVA) were performed to identify array components that correlated with blood type, race, and gender and to determine *p* values. Relationships with age were evaluated by Pearson correlation, Spearman correlation, and Kendall correlation. Representative array images can be found in the Supplementary Information, Figure S6. The full glycan array data can be found in the Supplementary Excel File.

## Additional Information

**How to cite this article**: Muthana, S. M. and Gildersleeve, J. C. Factors Affecting Anti-Glycan IgG and IgM Repertoires in Human Serum. *Sci. Rep*. **6**, 19509; doi: 10.1038/srep19509 (2016).

## Supplementary Material

Supplementary Information

Supplementary Table S1

## Figures and Tables

**Table 1:** Correlations of anti-glycan antibodies to blood group antigens.

Blood Group Anitgen	Training Set IgG (*p* values)	Validation Set1 IgG (*p* values)	Training Set IgM (*p* values)	Validation Set1 IgM (*p* values)
A tetra type 2-Sp - 17	2.54 × 10^−17^	5.52 × 10^−19^	6.01 × 10^−11^	4.48 × 10^−19^
Globo A - 09	4.15 × 10^−15^	1.82 × 10^−21^	7.59 × 10^−3^	2.45 × 10^−3^
A tetra type 1-Sp - 15	1.34 × 10^−14^	4.22 × 10^−20^	5.20 × 10^−5^	1.89 × 10^−10^
A tetra type 2-Sp - 05	2.19 × 10^−14^	5.17 × 10^−17^	8.58 × 10^−13^	3.16 × 10^−18^
A tetra type 2-Sp - 07	7.31 × 10^−14^	3.47 × 10^−17^	9.01 × 10^−12^	3.18 × 10^−18^
A tetra type 1-Sp - 05	3.11 × 10^−13^	5.52 × 10^−19^	2.84 × 10^−5^	9.11 × 10^−9^
2’F-A type 2-Sp - 13	2.04 × 10^−12^	2.63 × 10^−19^	3.64 × 10^−8^	2.32 × 10^−18^
2’F-A type 2-Sp - 05	2.09 × 10^−11^	6.65 × 10^−17^	1.02 × 10^−9^	2.81 × 10^−12^
BG-A1_penta_- 05	7.04 × 10^−9^	5.36 × 10^−14^	2.92 × 10^−2^	1.75 × 10^−8^
BG-A_tri_ -19	2.84 × 10^−7^	9.89 × 10^−13^	2.92 × 10^−4^	9.40 × 10^−8^
B tetra type 2-Sp - 05	1.74 × 10^−12^	2.18 × 10^−14^	6.00 × 10^−6^	1.31 × 10^−17^
B tetra type 2-Sp - 07	6.24 × 10^−12^	3.82 × 10^−15^	1.57 × 10^−5^	2.20 × 10^−15^
B tetra type 2-Sp - 20	1.24 × 10^−11^	4.01 × 10^−15^	2.81 × 10^−4^	2.07 × 10^−13^
2’F-B type 2-Sp - 15	4.90 × 10^−12^	5.10 × 10^−12^	2.19 × 10^−6^	3.85 × 10^−8^
2’F-B type 2-Sp - 07	8.14 × 10^−11^	5.51 × 10^−11^	2.21 × 10^−6^	2.61 × 10^−15^
B tetra type 1-Sp - 16	2.90 × 10^−9^	1.91 × 10^−16^	3.14 × 10^−2^	6.61 × 10^−8^
B tetra type 1-Sp - 04	1.24 × 10^−8^	4.22 × 10^−12^	1.24 × 10^−8^	1.07 × 10^−7^
BG-B_tri_ - 13	1.13 × 10^−5^	9.70 × 10^−13^	4.19 × 10^−2^	1.24 × 10^−7^

**Table 2:** Non-blood group antibody subpopulations that correlated with blood type.

Capture Glycan (abbreviation)	Structure or Name	Training Set (*p* values)	Validation Set (*p* values)
IgG			
alpha-Gal tetra - 17	Gala1-3Galb1-4GlcNAcb1-3Galb	2.05 × 10^−2^	1.84 × 10^−6^
alphaGal- 08	Gala1-3Galb1-4GlcNAc	3.18 × 10^−2^	7.49 × 10^−5^
Bdi-g - 16	Gala1-3Galb	9.26 × 10^−3^	8.35 × 10^−4^
Bdi -23	Gala1-3Gal	1.14 × 10^−2^	5.88 × 10^−3^
Gal3- 07	Gala1-3Galb1-4Gala	2.60 × 10^−3^	4.82 × 10^−3^
alphaGal-6-deoxy - 11	Gala1-3Galb1-4 6deoxy-GlcNAc	5.92 × 10^−2^	3.18 × 10^−6^
Gala3-type1 - 09	Gala1-3Galb1-3GlcNAc	3.02 × 10^−2^	1.13 × 10^−2^
Forssman Tetra-BSA - 05	GalNAca1-3GalNAcb1-3Gala1-4Galb	5.73 × 10^−3^	1.75 × 10^−3^
BSM	Bovine submaxillary mucin	7.53 × 10^−2^	5.48 × 10^−5^
IgM			
Ac-S-Tn(Ser)-S-G - 04	Ac-Ser-Ser(GalNAca)-Ser-Gly	3.82 × 10^−3^	9.16 × 10^−3^
Adi - 17	GalNAca1-3Galb	9.95 × 10^−3^	1.86 × 10^−2^
OSM (asialo)	asialo-Ovine submaxillary mucin	5.41 × 10^−3^	3.15 × 10^−2^
GalNAca1-6Galb - 22	GalNAca1-6Galb	2.01 × 10^−2^	2.30 × 10^−2^

**Table 3:** Anti-glycan antibody subpopulations that correlated with race.

Capture Glycan Abbreviation	Glycan structure	Training *p* value	Validation *p*value
LacNAc (dimeric)-Sp - 16	Galb1-4GlcNAcb1-3Galb1-4GlcNAcb	0.015	0.015
LNT-Sp - 15	Galb1-3GlcNAcb1-3Galb1-4GlcNAcb	0.011	0.022
3′GN-LacNAc (dimeric)-Sp - 06	GlcNAcb1-3Galb1-4GlcNAcb1-3Galb1-4GlcNAcb	0.013	0.022
3′GN-LacNAc (dimeric)-Sp - 14	GlcNAcb1-3Galb1-4GlcNAcb1-3Galb1-4GlcNAcb	0.017	0.032
6’Neu5Ac-LacNAc (dimeric)-Sp - 13	Neu5Aca2-6Galb1-4GlcNAcb1-3Galb1-4GlcNAcb	0.022	0.030
3′Neu5Ac-LacNAc (dimeric)-Sp - 13	Neu5Aca2-3Galb1-4GlcNAcb1-3Galb1-4GlcNAcb	0.055	0.064
