# Supplementary material for: Factors Affecting Anti-Glycan IgG and IgM Repertoires in Human Serum
Source: Sci Rep. 2016 Jan 19;6:19509. doi: 10.1038/srep19509 (PMC4726023; doi:10.1038/srep19509)
Supplement: Supplementary Information [file srep19509-s1.pdf]

Factors Affecting Anti-Glycan IgG and IgM Repertoires in Human Serum

Saddam M. Muthana[1,2] and Jeffrey C. Gildersleeve[1]

[1]Chemical Biology Laboratory, National Cancer Institute, NIH, 376 Boyles St., Frederick, MD 21702, USA

[2]Chemistry Department, College of Science & General Studies, Alfaisal University, Riyadh, KSA

Table of Contents
Page

Sample collection.. S2
Table SI.. S3
Figure S1.. S7
Figure S2.. S8
Figure S3 .... S9
Figure S4 .... S10
Figure S5.. S11
Figure S6.. S12

Sample collection

Sample processing and storage by both providers were similar except for how whole blood was clotted and centrifuged to obtain serum (room temperature for Valley Biomedical Products and Services versus 4 °C for Bioreclamation LLC). Briefly Blood was allowed to clot overnight at 4 °C (Bioreclamation) or at room temperature (Valley Biomedical Products and Services). The following day the serum is centrifuged, collected, and stored at -20 °C.

The validation set (n = 90) which included samples from both providers, 60 samples from Valley Biomedical Products and Services and 30 samples from Bioreclamation LLC was evaluated for whether differences in anti-glycan antibody signals due to possible differences in how the samples were collected. To assess the comparability of data obtained for serum from both providers, the average IgG and IgM intensities for each glycan in the array were calculated from each provider. The average IgG and IgM antibody signals were similar from both providers (Figure S5). The data suggests that sample collection and handling by the two providers in this study have minimal effect on anti-glycan antibody levels.

Table SI. List and description of sample characteristics from healthy individuals.

Sample #	Sex	Race	Age	Blood Type	Location
**1**	M	African-American	32	O	Florida
**2**	M	Hispanic	46	O	Florida
**3**	M	Caucasian	44	B	Florida
**4**	M	African-American	22	O	Florida
**5**	M	Caucasian	27	AB	Florida
**6**	M	African-American	43	O	Florida
**7**	M	African-American	25	O	Florida
**8**	M	Caucasian	29	AB	Florida
**9**	M	African-American	26	A	Florida
**10**	M	Hispanic	34	O	Florida
**11**	M	Hispanic	23	A	Florida
**12**	M	Caucasian	33	O	Florida
**13**	M	Caucasian	41	O	Florida
**14**	M	Caucasian	23	O	Florida
**15**	M	Caucasian	49	O	Florida
**16**	M	African-American	39	A	Florida
**17**	M	Hispanic	49	A	Florida
**18**	M	Caucasian	42	A	Florida
**19**	M	African-American	21	B	Florida
**20**	M	African-American	33	A	Florida
**21**	F	Caucasian	34	AB	Florida
**22**	F	Hispanic	34	A	Florida
**23**	F	African-American	50	A	Tennessee
**24**	F	African-American	38	AB	Florida
**25**	F	Hispanic	43	A	Florida
**26**	F	Hispanic	36	AB	Florida
**27**	F	Hispanic	21	AB	Florida
**28**	F	Caucasian	28	O	Tennessee
**29**	F	Caucasian	43	A	Tennessee
**30**	F	African-American	23	AB	Florida
**31**	F	Hispanic	42	AB	Florida
**32**	F	Hispanic	27	A	Florida
**33**	F	Caucasian	21	A	Tennessee
**34**	F	Hispanic	38	O	Florida
**35**	F	Hispanic	41	O	Florida
**36**	F	African-American	20	B	Tennessee
**37**	F	African-American	27	AB	Florida
**38**	F	Hispanic	41	AB	Tennessee
**39**	F	Hispanic	23	O	Florida

**40**	F	African-American	30	B	Tennessee
**41**	F	Caucasian	32	A	Tennessee
**42**	F	Caucasian	45	AB	Tennessee
**43**	F	Caucasian	36	O	Tennessee
**44**	F	African-American	43	AB	Florida
**45**	F	Caucasian	36	B	Tennessee
**46**	M	African-American	25	A	Florida
**47**	F	Hispanic	40	A	Florida
**48**	M	African-American	31	B	Florida
**49**	F	Hispanic	30	AB	Florida
**50**	M	Hispanic	38	O	Florida
**51**	F	African-American	40	B	Tennessee
**52**	M	Hispanic	44	O	Florida
**53**	F	Caucasian	32	A	Tennessee
**54**	M	Hispanic	24	A	Florida
**55**	F	Caucasian	42	A	Tennessee
**56**	M	Caucasian	41	B	Florida
**57**	F	Hispanic	22	O	Florida
**58**	M	Caucasian	26	O	Florida
**59**	F	African-American	43	O	Tennessee
**60**	M	African-American	40	O	Florida
**61**	F	Caucasian	45	O	Tennessee
**62**	M	Caucasian	43	O	Florida
**63**	F	Caucasian	23	AB	Florida
**64**	M	Caucasian	36	O	Florida
**65**	F	Caucasian	20	A	Tennessee
**66**	M	African-American	38	O	Florida
**67**	F	Caucasian	34	O	Tennessee
**68**	M	Hispanic	22	O	Florida
**69**	F	African-American	42	AB	Florida
**70**	M	African-American	40	A	Florida
**71**	F	Hispanic	39	AB	Florida
**72**	M	African-American	46	O	Florida
**73**	F	African-American	35	AB	Florida
**74**	M	Hispanic	45	O	Florida
**75**	F	Caucasian	33	A	Tennessee
**76**	M	Hispanic	30	A	Florida
**77**	F	Hispanic	37	A	Florida
**78**	M	African-American	23	O	Florida
**79**	F	Hispanic	31	AB	Florida
**80**	M	Caucasian	50	A	Florida

**81**	F	African-American	47	AB	Florida
**82**	M	African-American	20	B	Florida
**83**	F	African-American	38	AB	Florida
**84**	M	Hispanic	28	A	Florida
**85**	F	Caucasian	30	A	Tennessee
**86**	M	Caucasian	29	B	Florida
**87**	F	Hispanic	43	A	Florida
**88**	M	Caucasian	38	A	Florida
**89**	F	Caucasian	31	O	Tennessee
**90**	M	African-American	43	O	Florida
**91**	F	Hispanic	22	O	Florida
**92**	M	Caucasian	34	O	Florida
**93**	F	Hispanic	26	O	Florida
**94**	M	African-American	34	B	Florida
**95**	F	Caucasian	50	O	Tennessee
**96**	M	Hispanic	48	A	Florida
**97**	F	Caucasian	32	A	Tennessee
**98**	M	African-American	45	O	Florida
**99**	F	African-American	28	AB	Florida
**100**	M	Caucasian	21	O	Florida
**101**	F	African-American	44	A	Tennessee
**102**	M	Hispanic	36	A	Florida
**103**	F	African-American	43	AB	Florida
**104**	M	Hispanic	27	A	Florida
**105**	M	African-American	25	B	Florida
**106**	F	African-American	29	O	-
**107**	M	Hispanic	20	O	-
**108**	M	Hispanic	34	O	-
**109**	F	African-American	36	O	-
**110**	M	African-American	42	O	-
**111**	F	African-American	40	O	-
**112**	F	Hispanic	60	O	-
**113**	M	African-American	54	O	-
**114**	F	Hispanic	55	O	-
**115**	F	African-American	27	A	-
**116**	M	Caucasian	23	A	-
**117**	F	Caucasian	38	A	-
**118**	M	Hispanic	31	A	-
**119**	M	African-American	41	A	-
**120**	F	African-American	46	A	-
**121**	M	Caucasian	58	A	-

**122**	M	African-American	65	A	-
**123**	F	Hispanic	41	A	-
**124**	M	Caucasian	42	O	-
**125**	F	Hispanic	42	O	-
**126**	M	African-American	43	B	-
**127**	F	Hispanic	43	AB	-
**128**	F	Caucasian	43	AB	-
**129**	F	African-American	43	O	-
**130**	M	Caucasian	44	B	-
**131**	F	Caucasian	45	A	-
**132**	M	Hispanic	46	AB	-
**133**	M	Caucasian	49	O	-
**134**	M	Hispanic	49	AB	-
**135**	F	African-American	50	B	-

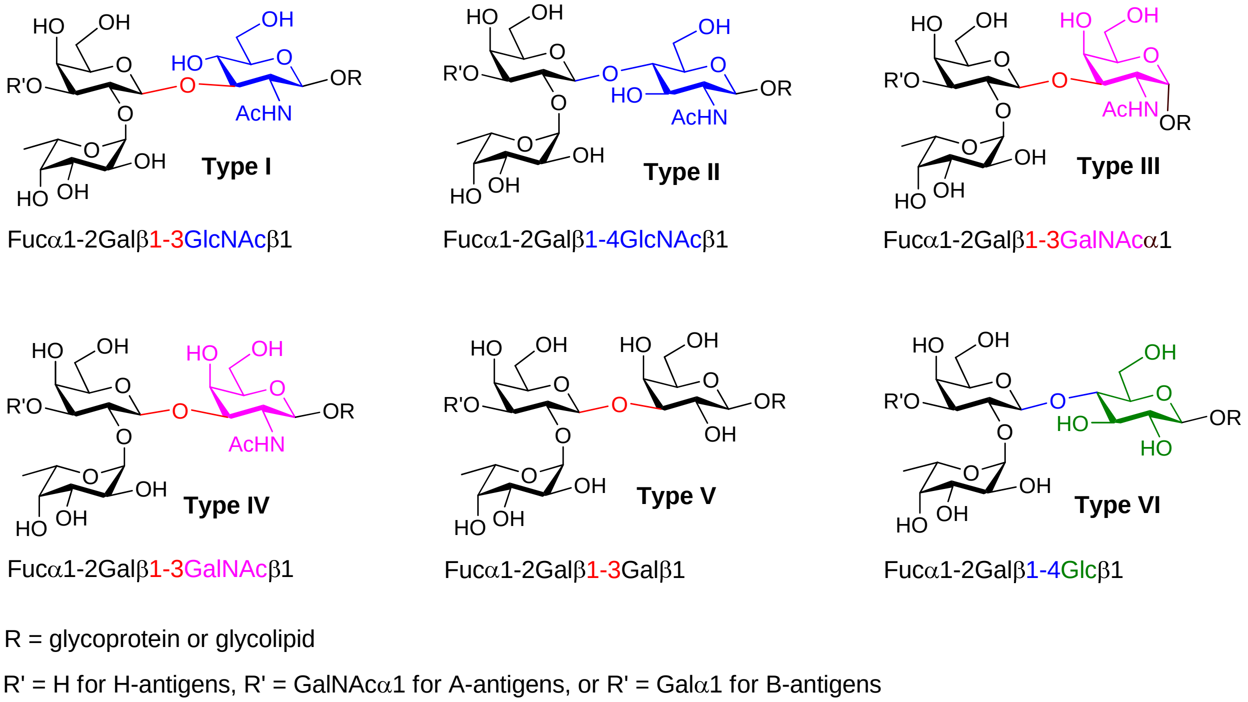

Figure S1. Structures of the different types of ABH blood group antigens (I-VI).

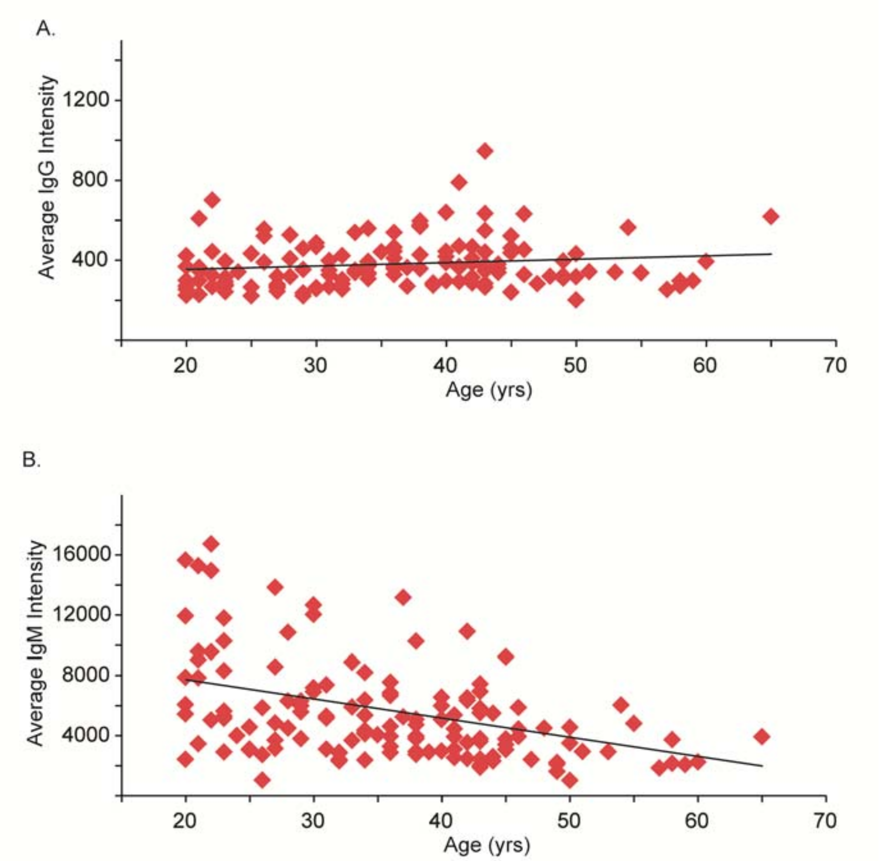

Figure S2. Age-related variations in anti-glycan antibodies in apparently healthy individuals (n = 135). (**A**) Scatter plot showing a relatively stable IgG antibody levels with age. (**B**) Scatter plot showing a drop in IgM antibody levels with age.

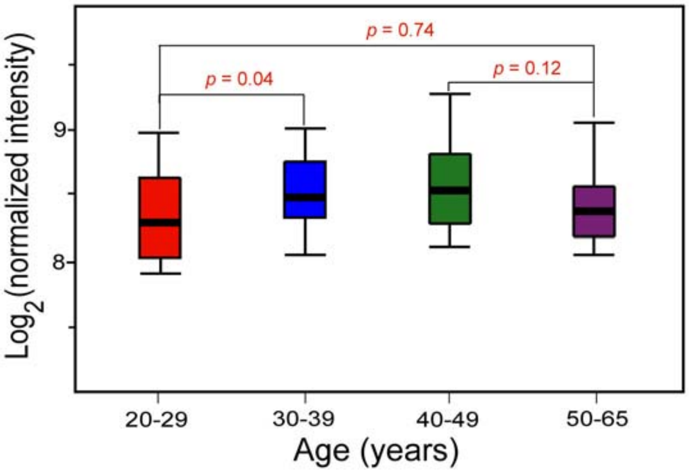

Figure S3. Box plots show minimum variation in anti-glycan IgG antibody levels with age when subjects were grouped every 10 years.

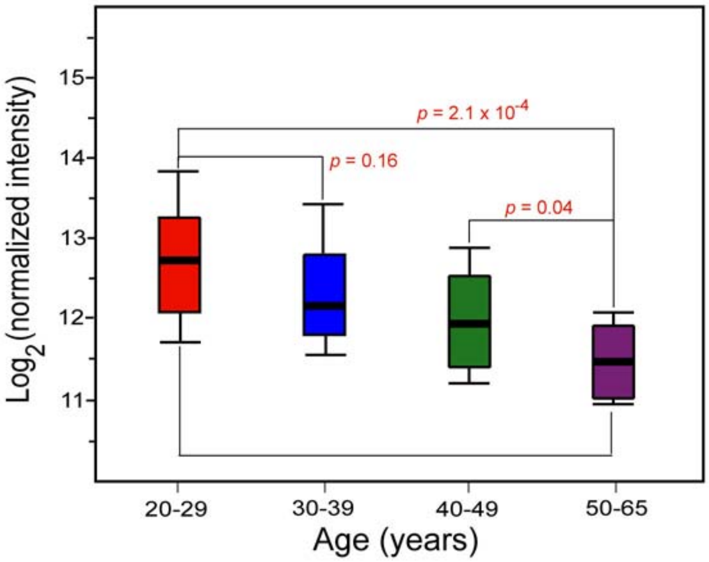

Figure S4. Box plots showing variations in anti-glycan IgM antibody levels with age when subjects were grouped every 10 years.

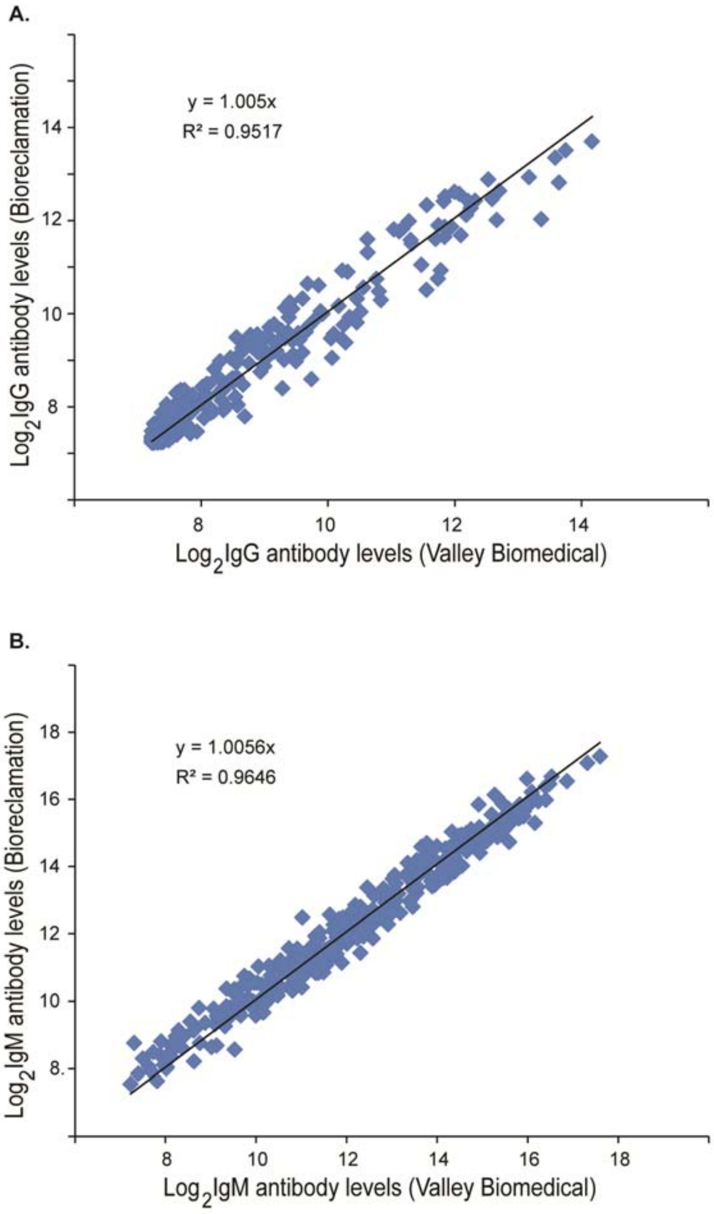

Figure S5. Scatter plots showing little variations in anti-glycan antibody levels due to sample collection and handling. (**A**) Level of IgG antibodies. (**B**) Level of IgM antibodies.

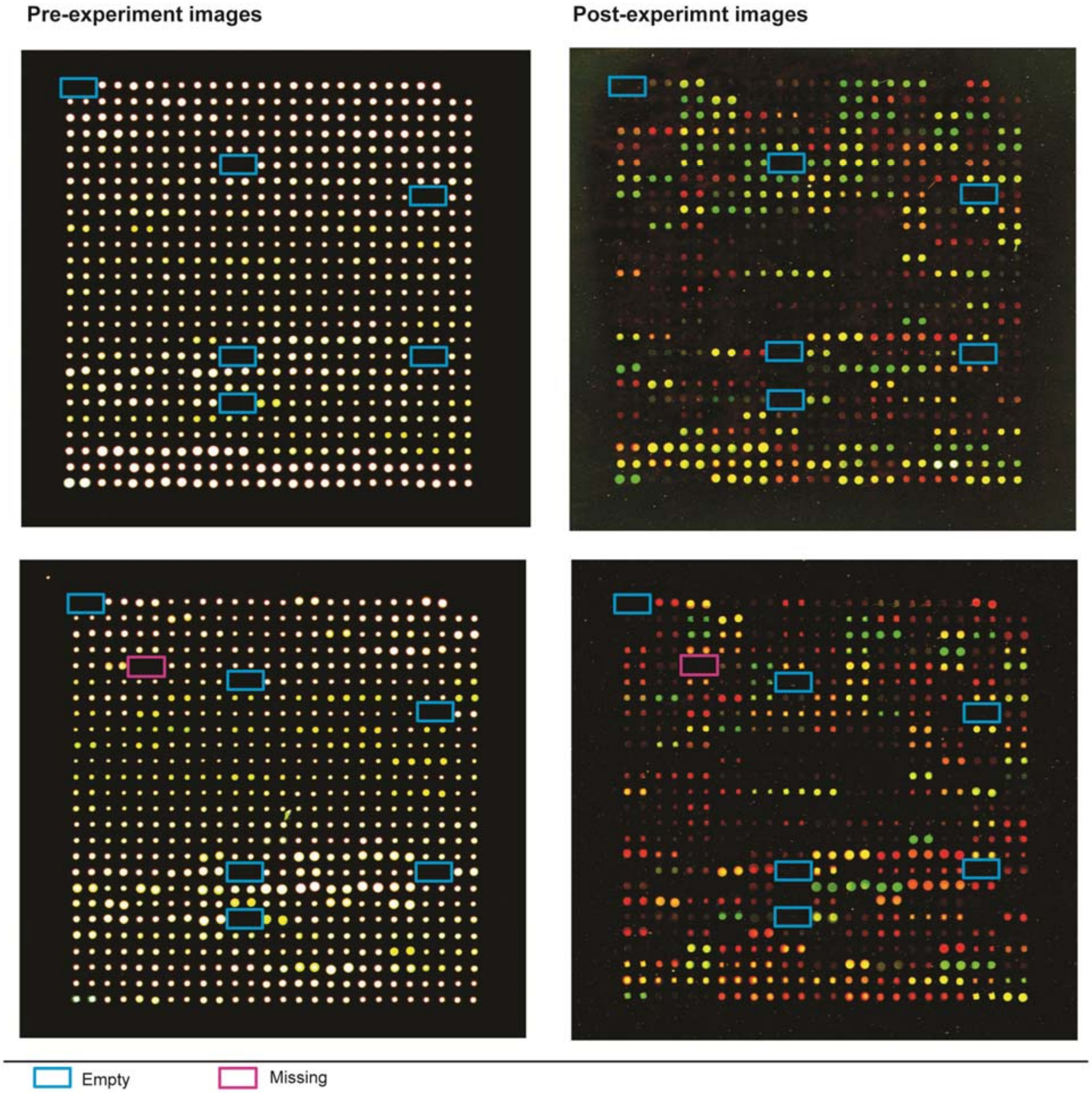

Figure S6. Representative examples of pre- and post-experimental array images. Signals in the Pre-experiment images are due to inclusion of a soluble fluorescent dye in the print buffer (the free acid of DyLight 649), which is washed away during blocking tp. Blue boxes indicate spots that were printed from empty wells, and pink boxes indicate spots that were missing during printing of the arrays.
